# The effectiveness of atrial fibrillation special clinic on oral anticoagulant use for high risk atrial fibrillation patients managed in the community

**DOI:** 10.1186/s12875-023-02004-w

**Published:** 2023-02-14

**Authors:** Ka Man Lau, To Fung Leung, Yim Chu Li, Catherine Xiao Rui Chen

**Affiliations:** 1 Department of Family Medicine and General Out-Patient Clinics, Kowloon Central Cluster of the Hospital Authority of Hong Kong, Kowloon, Hongkong; 2 Box Hill Superclinic, 810 Whitehorse Road, Box Hill, VIC 3128 Australia

**Keywords:** Atrial fibrillation, Oral anticoagulant, Cardiovascular disease risk factor, Primary care

## Abstract

**Background:**

Service gaps exist in oral anticoagulant (OAC) use among patients with atrial fibrillation (AF) in primary care. The purpose of this study was to explore the clinical effectiveness of a community dwelling Atrial Fibrillation Special Clinic (AFSC) run by primary care physicians by evaluating its impact on OAC use and the control of modifiable cardiovascular disease (CVD) risk factors in high risk AF patients.

**Method:**

Quasi-experimental study was conducted in AFSC run by public primary care physicians in Hong Kong. Study subjects were high risk AF patients with CHA_2_DS_2_-VASc scores ≥ 2, who had been followed up (FU) at AFSC for at least one year from 01 August, 2019 to 31 October, 2020. OAC usage and modifiable CVD risk factor control were compared before and after one year of FU at AFSC. Drug-related adverse events, emergency attendance or hospitalisation episodes, survival and mortality rates after one year FU at AFSC were also reviewed.

**Results:**

Among the 299 high risk AF patients included in the study, significant increase in OAC use was observed from 58.5% at baseline to 82.6% after one year FU in AFSC (*P* < 0.001). Concerning CVD risk factor control, the average diastolic blood pressure level was significantly reduced (*P* = 0.009) and the satisfactory blood pressure control rate in non-diabetic patients was markedly improved after one year FU (*P* = 0.049). However, the average HbA1c and LDL-c levels remained static. The annual incidence rate of ischaemic stroke/systemic embolism was 0.4%, intra-cranial haemorrhage was 0.4%, major bleeding episode was 3.2% and all-cause mortality was 4.3%, all of which were comparable to reports in the literature.

**Conclusion:**

AFSC is effective in enhancing OAC use and maintaining optimal modifiable CVD risk factor control among high risk AF patients managed in primary care setting, and therefore may reduce AF-associated morbidity and mortality in the long run.

## Introduction

Atrial fibrillation (AF) is a common type of arrhythmia encountered in primary care and is a cause of significant morbidity and mortality [1, 2]. Globally, 33.5 million patients had AF in 2010 and AF affects approximately 1% of the population in Hong Kong. With the aging of the population, the number of new AF cases was estimated to be 4.7 million per year [3], with greater prevalence in elderly individuals and in patients with comorbidities [4, 5].

Patients with AF have five-fold increased risk of stroke compared with non-AF patients [6], and the use of oral anticoagulation (OAC) significantly reduced the risk of stroke in AF patients [7]. Therefore, OACs are an integral part of AF management to prevent the thromboembolic events.

Strict control of cardiovascular disease (CVD) risk factors is also an essential part of AF management. For example, studies have shown that early detection and optimal control of modifiable CVD risk factors such as hypertension (HT), diabetes mellitus (DM), obesity, congestive heart failure (CHF), myocardial infarction, valvular heart disease, smoking and alcohol consumption etc. could all effectively prevent the progression of AF and reduce AF related morbidity and mortality [8–12].

Despite all this evidence, service gaps exist in AF management, particularly in the persistently low utilization rate of OACs among AF patients [13–15]. For example, a study in U.S. showed that only 11–78.8% of indicated AF patients were put on OACs [16], while a study in China found that a total of 35.6% of indicated AF patients had received OACs and only 11.1% of them were using Novel OACs (NOACs) [17]. Similarly, a local study conducted in hospital setting revealed that only 23% of high risk AF patients had received OACs [18]. At this moment, there is no information on OAC use among AF cases managed in primary care setting and their CVD risk factor control. To address all these service gaps, the AF Special Clinic (AFSC) was established in the Department of Family Medicine and General Outpatient Clinics (Dept. of FM and GOPCs) of Kowloon Central Cluster of Hospital Authority of Hong Kong (HAHK) in June 2019. The aim of setting up this clinic is to provide holistic and comprehensive management to AF patients in the community. This study tried to explore the clinical effectiveness of AFSC by evaluating its impact on OAC use and the control of CVD risk factors among high risk AF patents managed by primary care physicians. We believe that AFSC would help enhance OAC utilization and improve CVD risk factor control, and hence reducing AF related mortality in the long run.

## Methods

### Study design

A quasi-experimental, pre- and post-test study design was used to compare the outcome parameters.

### Definition of different risks of AF in this study

The CHA_2_DS_2_-VASc score (Congestive heart failure, HT, Age ≥ 75 years [doubled], DM, prior Stroke or transient ischemic attack [doubled], Vascular disease, Age 65–74 years, and Sex category [female]) is a well-validated risk-stratification score for predicting stroke events in patients with AF [19]. According to the AF management guidelines from the European Society of Cardiology [20], the CHA_2_DS_2_-VASc score should be calculated for all AF cases to stratify their stroke risk. If the score ≥ 2, the patient is considered as a ‘high risk’ AF patient and OAC is recommended. If the score is 0 in males or 1 in females, the CVD risk is low and therefore no OAC therapy is recommended. In males whose score is = 1, OACs may be considered, and people's values and preferences should be considered [21].

### Study subjects

All high risk AF patients coded by the International Classification of Primary Care 2^nd^ version (ICPC-2)-code of “K78” (atrial fibrillation), whose CHA_2_DS_2_-VASc score was ≥ 2, had been followed up (FU) for at least one year at 5 AFSCs of HAHK from 01 August, 2019 to 31 October, 2020.

AF patients were excluded if they had contraindications to NOAC therapy including known hypersensitivity, clinically significant active bleeding, significant inherited or acquired bleeding disorder, hepatic disease associated with coagulopathy, significant risk of major bleeding (such as current or recent gastrointestinal ulceration, presence of malignant neoplasms at high risk of bleeding, recent brain or spinal injury/surgery, recent intracranial haemorrhage), severe renal impairment (calculated creatine clearance < 30 mL/min for dabigatran and < 15 mL/min for apixaban), pregnancy and breastfeeding. Patients who defaulted FU at AFSC, had incomplete data, transferred to be cared for by other specialists or were certified dead during the study period were excluded from the final data analysis.

### Management at AFSC

The attending doctors at AFSC were experienced Family Medicine (FM) specialists who had received training on AF management via standardized educational talk. Patient epidemiological characteristics such as age, gender, smoking status, drinking status, comorbidities including HT, DM and CHF, past history of ischaemic heart disease (IHD), stroke/transient ischaemic attack (TIA) or intra-cranial haemorrhage (ICH) and type of AF (non-valvular, which confirmed by physical examination and previous echocardiography result) were reviewed. The CHA_2_DS_2_-VASc score and HAS-BLED score (Hypertension, Abnormal renal and liver function, Stroke, Bleeding tendency, Labile INRs, Elderly, Drugs or Alcohol), which predicts bleeding risk were calculated and documented. Baseline blood tests including complete blood picture, clotting profile, serum creatinine, alanine transaminase, glucose, HbA1c and lipid profile were checked. The estimated glomerular filtration rate (eGFR) was calculated by using the Chronic Kidney Disease Epidemiology Collaboration (CKD-EPI) equation [22].

With the introduction of NOACs to the Drug Formulary of GOPCs in HAHK in July 2019, AF patients whose CHA_2_DS_2_-VASc score was ≥ 5 could obtain NOACs for free in the HA Pharmacy. For those whose CHA_2_DS_2_-VASc score was between 2–4, the patients had to purchase the NOAC as a self-financed item (SFI) from community pharmacy. Two types of NOACs were available in AFSC in KCC, Dabigatran and Apixaban. Patients could also choose other NOACs as SFIs, such as rivaroxaban or endoxaban. If AF patients were found to have moderate to severe mitral valve stenosis or had undergone valvular replacement therapy, they were referred to a specialist setting for warfarin treatment. The potential risks and benefits of anticoagulation therapy were thoroughly discussed with the patients by the attending FM doctor. Updated international guidelines and appropriate local therapeutic instructions were also available on our department website. At each FU visit at AFSC, patient medication adherence and adverse effects were assessed. Blood test results, accident and emergency department (AED) admission or hospitalizations were also documented.

### Data collection

The list of patients fulfilling the inclusion criteria was retrieved from the Clinical Data Analysis and Reporting System (CDARS) of HA. Patient age, gender, smoking status and alcohol status were retrieved from the Clinical Management System (CMS) of HA. Their clinic blood pressure (BP) level on the first AFSC attendance and after one year FU were collected. The biochemical parameters including HbA1c and LDL-c levels before AFSC recruitment and after one year FU at AFSC were compared. Their AED attendance, hospitalization records and mortality data during the study period were also retrieved from the CMS.

### Outcome measures

The *primary outcomes* include the following:

1. Total number of patients who agreed to NOAC treatment after recruitment in the AFSC, and
2. Modifiable CVD risk factor control, in terms of BP, HbA1c and LDL-c levels at baseline and after one year FU.
  For HT patients without DM, BP < 140/90 mmHg was defined as satisfactory control
  For HT patients with DM, BP < 130/80 mmHg was defined as satisfactory control
  For DM patients, HbA1c < 7% was defined as satisfactory glycaemic control
  For patients without history of CVD, LDL-c < 2.6 mmol/L was defined as satisfactory lipid control
  For patients with history of CVD, LDL-c < 1.8 mmol/L was defined as satisfactory lipid control

The *secondary outcomes* after one year FU include the following:

1. Drug-related adverse events
2. Major bleeding and non-major bleeding episodes
3. Stroke or systemic embolism events
4. AED attendance or hospitalisation episodes
5. Survival and mortality rates

Major bleeding episodes (MBEs) were defined per the International Society on Thrombosis and Hemostasis (ISTH) criteria as one of the following [23]: fatal bleeding, and/or symptomatic bleeding in a critical area or organ, such as intracranial, intraspinal, intraocular, retroperitoneal, intra-articular or pericardial, or intramuscular with compartment syndrome, and/or clinically overt bleeding with a decrease in the haemoglobin level of ≥ 2 g/dl or transfusion of ≥ 2 units of packed red cells. Any reported bleeding episode that did not meet the criteria for major bleeding was defined as a non-major bleeding episode (NMBE).

The project terminated when the AF patient had completed one year FU at AFSC or developed serious adverse effects related to intervention with supportive evidence.

### Sample size calculation

Based on the local study of AF prevalence and NOAC utilization [18, 24] as well as the level of significance (α = 0.05), the power of the test (β = 0.2 power of the test 80%) and the effect size (*d* = 0.5), the minimum sample size is 283. To allow room for case exclusion and assume a 15% dropout rate, 325 people were recruited.

### Statistical analysis

All data were entered and analyzed using computer software (Windows version 23.0; SPSS Inc, Chicago [IL], US). Patient characteristics were described using proportions for categorical variables and means with standard deviations for continuous variables. Baseline characteristics are presented as percentages for categorical variables and mean ± standard deviation (SD) for continuous variables. The Chi-square test was used for univariate comparisons of categorical variables between groups. Student’s t test was used for continuous variables. All statistical tests were two sided, and a *P* value of less than 0.05 was considered statistically significant.

### Ethical approval

The study was approved by the Research Ethics Committee of Kowloon Central Cluster of Hospital Authority of Hong Kong, and the approval number was KC/KE-19–0143/ER-3.

## Results

In total, 325 high risk AF patients had attended AFSC during the study period, among which 194 patients had already taken NOAC whereas 131 patients had not. After thorough discussion with the attending FM specialist doctor in AFSC, 72 patients who did not take NOAC before agreed to start NOAC, whereas only 59 patients still declined it. Among the NOAC group, a total of 19 patients were excluded after a one-year FU, with 6 FU in the Specialist Out-patient Clinic, 2 defaulted FU and 11 patients died. In the non-NOAC group, 7 patients were excluded, with 2 defaulted FU, 3 cases with incomplete data and 2 patients died.

Among the 11 patients who died in the NOAC group, 1 patient died of ICH at 6 months after initiation of NOAC with incidence rate of 0.4% and 1 patient died of ischaemic stroke who had already taken NOAC prior to attending AFSC, with an incidence rate 0.4%. The causes of the other 9 deaths were non-NOAC related including pneumonia, IHD and cancer. The one-year all-cause mortality rate in the NOAC group was 4.3%.

Regarding the 2 patients who died in the non-NOAC group, both died of pneumonia, with one-year all-cause mortality rate of 3.7%, which was not significant (*P* = 0.85) compared with the NOAC group.

**Fig. 1:**
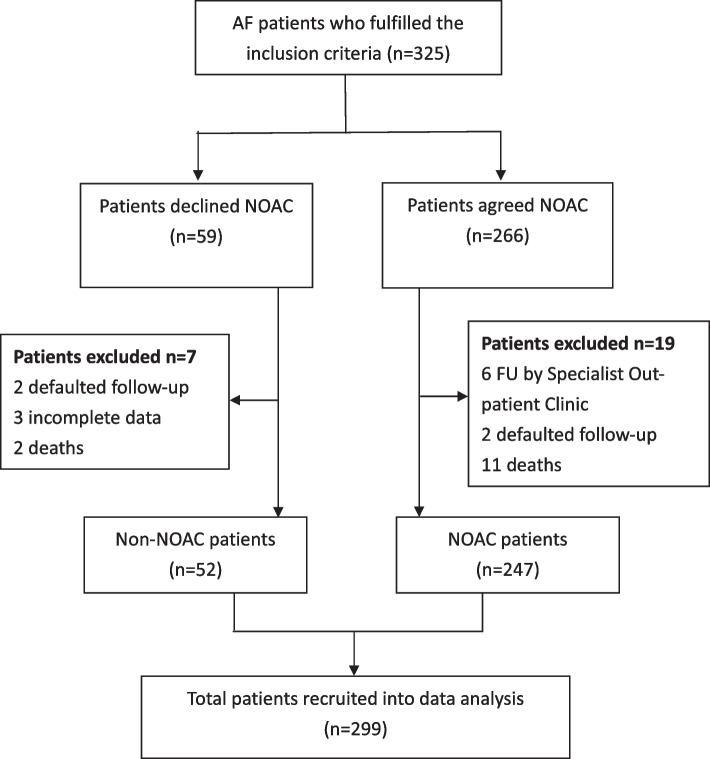
Flow chart of case recruitment at AFSC during the study period. AF, atrial fibrillation; AFSC, atrial fibrillation special clinic; NOAC, novel oral anticoagulant

After case exclusion, a total of 299 cases including 247 patients on NOAC and 52 patients who declined NOAC were included in the final data analysis. The flowchart of case recruitment for this study is summarized in Fig. 1.

Among the 299 patients included in the data analysis, their mean age was 82.5 ± 7.4 years, with 87% over 75 years, 12% 65 to 74 years old, and only 1% younger than 65 years old. Two hundred (66.9%) patients were female, and 99 (33.1%) were male. The majority of patients were non-smoker and non-drinker with 82.3% and 97.6% respectively. The mean CHA_2_DS_2_-VASc score was 5.38 (± 0.95), with 90.3% of patients having CHA_2_DS_2_-VASc score ≥ 5 and 9.7% having CHA_2_DS_2_-VASc score 2–4, and the mean HAS-BLED score was 1.70 (± 0.69).

Regarding the AF related risk factors, there were 288 (96.3%) patients with HT, 161 (53.8%) patients with DM, 52 (17.4%) patients with CHF, 49 (16.4%) patients with IHD and 150 (50.2%) patients with previous history of stroke/TIA. A total of 156 (52.2%) patients had chronic kidney disease (CKD) with eGFR < 60 mL/min/1.73 m^2^. Table 1 summarizes the demographic characteristics, comorbidities and NOAC use profile of AF patients FU at AFSC.

**Table 1:** Demographic characteristics, comorbidities and NOAC profiles of AF patients in the study

Characteristics	Total Number (*n* = 299)
Age (years)	82.5 (± 7.4)
< = 64 years	3 (1.0)
65-74 years	36 (12.0)
> = 75 years	260 (87.0)
Gender	
Female	200 (66.9)
Male	99 (33.1)
Smoking status	
Non-smoker	246 (82.3)
Ex-smoker	44 (14.7)
Smoker	9 (3.0)
Drinking status	
Non-drinker	292 (97.6)
Ex-drinker	2 (0.7)
Drinker	3 (1.0)
Social-drinker	2 (0.7)
Comorbidities	
Hypertension	288 (96.3)
Diabetes mellitus	161 (53.8)
Congestive heart failure	52 (17.4)
Ischaemic heart disease	49 (16.4)
Previous stroke/TIA	150 (50.2)
Previous intra-cranial haemorrhage	3 (1.0)
Renal function	
eGFR ≥ 60 mL/min/1.73 m^2^	143 (47.8)
eGFR 30–59 mL/min/1.73 m^2^	150 (50.2)
eGFR < 29 mL/min/1.73 m^2^	6 (2.0)
Scores	
CHA_2_DS_2_-VASc score	5.38 (± 0.95)
0–1	0
2–4	29 (9.7)
≥5	270 (90.3)
HAS-BLED score	1.70 (± 0.69)
NOAC at baseline	175 (58.5)
Dabigatran	90 (30.1)
Apixaban	52 (17.4)
Rivaroxaban	25 (8.4)
Warfarin	8 (2.7)
non-NOAC at baseline	124 (41.5)
Aspirin	115 (38.5)
Clopidogrel	3 (1.0)
None	6 (2.0)

Data are shown as mean (± standard deviation) or number of cases (%)

### Primary outcomes

AF patients who agreed to NOAC use after visiting the AFSC showed a statistically significant increase from 58.5% at baseline to 82.6% (*P* < 0.001) as shown in Table 2. Among them, 105 (35.1%) patients were prescribed dabigatran, 139 (46.5%) were on apixaban, and 3 (1%) were on rivaroxaban as SFI.

**Table 2:** Comparison of NOACs utilization in AF patients before and after recruited at AFSC (*n* = 299)

Variables	Before AFSC	After AFSC	*P*-value^#^
**NOAC**	175 (58.5)	247 (82.6)	** < 0.001**
CHA_2_DS_2_-VASc score 2–4	11 (3.7)	18 (6.0)	
CHA_2_DS_2_-VASc score ≥ 5	164 (54.8)	229 (76.6)	
Dabigatran	90 (30.1)	105 (35.1)	
Apixaban	52 (17.4)	139 (46.5)	
Rivaroxaban	25 (8.4)	3 (1.0)	
Warfarin	8 (2.7)	0	
**non-NOAC**	124 (41.5)	52 (17.4)	** < 0.001**
CHA_2_DS_2_-VASc score 2–4	18 (6.0)	11 (3.7)	
CHA_2_DS_2_-VASc score ≥ 5	106 (35.5)	41 (13.7)	
Aspirin	115 (38.5)	36 (12.0)	
Clopidogrel	3 (1.0)	3 (1.0)	
None	6 (2.0)	13 (4.3)	

Data are shown as number of patients (%)

^#^ For comparison of AF patients before and after recruited at AFSC by Chi-squared test

Table 3 summarizes modifiable CVD risk factor control in patients on NOACs at baseline and after one year FU. Among the 236 patients with HT, their average systolic BP (SBP) was 128.1 (± 13.3) mmHg and their average diastolic BP (DBP) was 71.0 (± 11.5) mmHg. After one year FU, SBP remained static at 126.9 (± 10.9) mmHg (*P* = 0.30), and the DBP was significantly decreased to 68.3 (± 10.6) mmHg (*P* = 0.009). For hypertensive AF patients without DM, 81.7% (*n* = 89) patients achieved satisfactory BP control and the rate was further increased to 90.8% (*n* = 99) after one year FU (*P* = 0.049). In hypertensive patients with DM, the BP control rate remained static after one year FU (*P* = 0.52).

**Table 3:** Modifiable CVD risk factor control in NOAC group at baseline and after one year FU

Variables	At baseline	After 12 months	*P*-value^§^
Hypertension (*n* = 236)			
SBP mmHg	128.1 (± 13.3)	126.9 (± 10.9)	0.30
DBP mmHg	71.0 (± 11.5)	68.3 (± 10.6)	***0.009***
1) without DM (*n* = 109)			
Number of patients with satisfactory control	89 (81.7)	99 (90.8)	***0.049***
2) with DM (*n* = 127)			
Number of patients with satisfactory control	76 (59.8)	81 (63.8)	0.52
Diabetes mellitus (*n* = 130)			
HbA1c %	6.68 (± 0.71)	6.65 (± 0.77)	0.71
Number of patients with satisfactory control	89 (68.5)	97 (74.6)	0.27
Hyperlipidemia (*n* = 247)			
LDL-c mmol/L	1.70 (± 0.55)	1.62 (± 0.52)	0.08
1) without history of CVD (*n* = 82)			
Number of patients with satisfactory control	77 (93.9)	79 (96.3)	0.72
2) with history of CVD (*n* = 165)			
Number of patients with satisfactory control	109 (66.1)	125 (75.8)	0.05

Data are shown as mean (± standard deviation) and number of patients (%)

Among the 130 AF patients comorbid with DM, their average HbA1c level (6.68% versus 6.65%) and satisfactory glycaemic control rate remained static from baseline to one year after FU (*P* = 0.71 and *P* = 0.27 respectively). The average LDL-c level at baseline and one year after FU was also comparable (1.70 mmol/L versus 1.62 mmol/L, *P* = 0.08) and subgroup analysis showed that the LDL-c control rate remained static in both the with or without history of CVD groups, *P* = 0.05 and *P* = 0.72 respectively.

### Secondary outcomes

Upon completion of the 12-month FU, a total of 12 bleeding episodes were observed, of which 8 were MBE at a rate of 3.2%/year, and 4 (1.6%/year) were NMBE.

The 8 patients with MBE were due to gastrointestinal (GI) bleeding, within which 3 patients were put on NOAC < 3 months (1.2%), 2 patients < 6 months (0.8%), 2 patients < 12 months (0.8%) and 1 patient was put on NOAC > 1 year (0.4%). Of the 4 patients who suffered from NMBE, 3 patients reported haematuria and 1 patient had haemoptysis.

We also observed total 65 AED attendance/ hospitalisation events with incidence rate 26.3%. Causes of admission included pneumonia, CHF, IHD, atypical chest pain, syncope, fall with or without fracture and cancer. 2 patients complained of non-specific general discomfort, tiredness and muscle discomfort after taking NOACs and they consequently declined to use NOACs. There were no serious adverse effects observed.

## Discussion

In our study, there was a significant increase in NOAC utilization after the AF cases were enrolled to be cared for in the AFSC. After one year FU at AFSC, 82.6% of AF patients had been put on NOAC, a rate that was significantly higher than those reported in the literature. Indeed, there are many barriers to initiating OAC treatment among AF patients. For example, overestimation of the bleeding risk and disadvantages associated with advanced age, such as fall risk etc., are other well-known obstacles [25]. Furthermore, lack of reversal agents may also affect patients’ decisions to use NOACs [26]. The reasons contributing to the satisfactory utilization rate of NOACs in our study were multi-factorial. First, most of the AF cases referred to AFSC were of high risk or very high risk groups, therefore they were more willing to try NOAC after discussion with the doctor. Second, with the availability of NOACs including apixaban and dabigatran at AFSC of HAHK since March 2019 and the implementation of the HAHK policy that AF patients whose CHA_2_DS_2_-VASc score is ≥ 5 can be provided with NOACs for free have helped eased the financial difficulty of many high risk AF patients, many of whom otherwise have to purchase the NOACs as SFIs before this exercise. Based on these positive results, we would like to propose to the Hong Kong government that free NOACs should be provided for all high risk AF patients whose CHA_2_DS_2_-VASc score is ≥ 2, although balancing the use of public resources and costs is also important. Third, the attending doctors at AFSC are experienced FM specialists who are more skillful in AF management. They provided a comprehensive assessment of AF patients’ background characteristics and comorbidities, and provided a thorough explanation and education of NOAC use to AF patients.

In recent years, more evidence has supported an integrated multidisciplinary approach with treatments and management of modifiable CVD risk factors and underlying conditions could slow progression and improve the outcomes of AF [27]. Greater reduction in BP and better glycaemic control and lipid profiles were associated with decreased AF frequency, duration and symptoms [12]. AFSC aimed to provide comprehensive care with treatment and tailored information about advice and education on risk factor management to AF patients by targeting their underlying medical conditions. Our study showed a reduction in average DBP and more non-DM hypertensive patients with satisfactory BP control after FU in AFSC. Although HbA1c and LDL-c levels showed no significant change after one year FU, their satisfactory control rate remained consistently high from baseline until one year FU. Therefore, AFSC could help AF patients maintain optimal CVD risk factor control, which may subsequently prevent the development of AF related complications.

The safety and efficacy of NOACs for the general population have been well demonstrated by different clinical trials in recent years. For example, a retrospective observational study found that both apixaban and dabigatran had lower incidences of ischaemic stroke (1.3–1.4%) and MBE (3.6%) than warfarin [28]. Our study showed comparable results with the literature, with an annual MBE incidence of 3.2%. The lower incidence of ischaemic stroke (0.4%) of our study might be due to the strict and satisfactory CVD risk factor control among AF patients managed at AFSC. Concerning the mortality rate, our study showed that the all-cause mortality rate of NOAC use after one year was 4.3%, which was also consistent with findings from the UK which showed an all-cause mortality rate of 4% in a large cohort study [29]. Therefore, the use of NOACs in AFSC was proven to be safe and effective with comparable stroke risk, bleeding risk and mortality rate to findings in the literature.

This study is the first study to assess OAC use and CVD risk factor control among high risk AF patients managed by primary care physicians. It has provided important background information on OAC use in the public primary care setting and helps to identify service gaps and direct future service enhancement strategies. In addition, all parameters including BP, HbA1c and LDL level were based on data of objective assessment retrieved from the CMS, thus recall bias or data entry bias had been minimized. Having said so, this study has several limitations. First, as this study was performed in public general out-patient clinics of a single cluster in HA, selection bias might exist. The results from this study may not be applicable to the private sector or secondary care setting. In addition, most of the AF cases assessed at AFSC had a higher CHA2DS2-VASc score of ≥ 5 (90.3%) due to HAHK Drug Formulary revamping exercise, which might have further confounded the findings of the study. The much higher NOAC utilization rate achieved at AFSC may not be comparable to other settings where most AF patients had a lower CHA_2_DS_2_-VASc score of 2–4. Second, due to the intrinsic limitations of the study design, the quasi experimental design without a control group, acausal temporal relationship could be established. Third, the one-year FU duration may not be long enough to assess the long-term effect of NOAC use among AF patients. In this regard, a randomized-control study design with a control group, and a longer FU study (more than one year) would help evaluate the efficacy of AFSC more comprehensively. Furthermore, a study of underlying obstacles to OAC prescription and subgroup analysis of the safety and effectiveness of NOACs may help physicians make more sensible clinical decision.

## Conclusion

AFSC is effective in enhancing OAC use and maintaining optimal modifiable CVD risk factor control among high risk AF patients managed in primary care setting. With a much higher rate of OAC use and better CVD risk factor control, it is postulated that AF associated morbidities and mortality will be reduced in the long run.

## Data Availability

The datasets generated and/or analysed during the current study are not publicly available to protect the confidentiality of participants’ data but are available from the corresponding author upon reasonable request.

## Abbreviations

AED: Accident and Emergency Department
AF: Atrial Fibrillation
BP: Blood Pressure
CMS: Clinical Management System
CVD: Cardiovascular disease
DM: Diabetes Mellitus
DBP: Diastolic BP
eGFR: Estimated Glomerular Filtration Rate
FU: Follow-up
HbA1c: Haemoglobin A1c
HA: Hospital Authority
HT: Hypertension
ICH: Intra-Cranial Haemorrhage
IHD: Ischaemic Heart Disease
KCC: Kowloon Central Cluster
LDL-c: Low-Density Lipoprotein-c
NOAC: Novel oral anticoagulant
OAC: Oral anticoagulant
SFI: Self-Financed Item
SOPC: Specialist Outpatient Clinic
SBP: Systolic BP
TIA: Transient Ischaemic Attack
VKA: Vitamin K Antagonist

## References

[1] Benjamin EJ, Wolf PA, D'Agostino RB, Silbershatz H, Kannel WB, Levy D (1998). Impact of atrial fibrillation on the risk of death: the Framingham Heart Study. Circulation.

[2] Staerk L, Sherer JA, Ko D, Benjamin EJ, Helm RH (2017). Atrial Fibrillation: Epidemiology, Pathophysiology, and Clinical Outcomes. Circ Res.

[3] Chugh SS, Havmoeller R, Narayanan K (2014). Worldwide epidemiology of atrial fibrillation: a Global Burden of Disease 2010 Study. Circulation.

[4] Ball J, Carrington MJ, McMurray JJ, Stewart S (2013). Atrial fibrillation: profile and burden of an evolving epidemic in the 21st century. Int J Cardiol.

[5] Kannel WB, Wolf PA, Benjamin EJ, Levy D (1998). Prevalence, incidence, prognosis, and predisposing conditions for atrial fibrillation: population-based estimates. Am J Cardiol.

[6] Saposnik G, Gladstone D, Raptis R, Zhou L, Hart RG, Investigators of the Registry of the Canadian Stroke Network (RCSN) and the Stroke Outcomes Research Canada (SORCan) Working Group (2013). Atrial fibrillation in ischemic stroke: predicting response to thrombolysis and clinical outcomes. Stroke..

[7] Hart RG, Pearce LA, Aguilar MI (2007). Meta-analysis: antithrombotic therapy to prevent stroke in patients who have nonvalvular atrial fibrillation. Ann Intern Med.

[8] Huxley RR, Lopez FL, Folsom AR (2011). Absolute and attributable risks of atrial fibrillation in relation to optimal and borderline risk factors: the Atherosclerosis Risk in Communities (ARIC) study. Circulation.

[9] Wyse DG, Van Gelder IC, Ellinor PT (2014). Lone atrial fibrillation: does it exist?. J Am Coll Cardiol.

[10] Violi F, Soliman EZ, Pignatelli P, Pastori D (2016). Atrial Fibrillation and Myocardial Infarction: A Systematic Review and Appraisal of Pathophysiologic Mechanisms. J Am Heart Assoc..

[11] Santhanakrishnan R, Wang N, Larson MG (2016). Atrial Fibrillation Begets Heart Failure and Vice Versa: Temporal Associations and Differences in Preserved Versus Reduced Ejection Fraction. Circulation.

[12] Pathak RK, Middeldorp ME, Lau DH (2014). Aggressive risk factor reduction study for atrial fibrillation and implications for the outcome of ablation: the ARREST-AF cohort study. J Am Coll Cardiol.

[13] Wong CW (2016). Anticoagulation for stroke prevention in elderly patients with non-valvular atrial fibrillation: what are the obstacles?. Hong Kong Med J.

[14] Giustozzi M, Agnelli G, Quattrocchi S (2020). Rates and Determinants for the Use of Anticoagulation Treatment before Stroke in Patients with Known Atrial Fibrillation. Cerebrovasc Dis Extra.

[15] Soo Y, Chan N, Leung KT (2017). Age-specific trends of atrial fibrillation-related ischaemic stroke and transient ischaemic attack, anticoagulant use and risk factor profile in Chinese population: a 15-year study. J Neurol Neurosurg Psychiatry.

[16] Marzec LN, Wang J, Shah ND (2017). Influence of Direct Oral Anticoagulants on Rates of Oral Anticoagulation for Atrial Fibrillation. J Am Coll Cardiol.

[17] Liu T, Yang HL, Gu L (2020). Current status and factors influencing oral anticoagulant therapy among patients with non-valvular atrial fibrillation in Jiangsu province, China: a multi-center, cross-sectional study. BMC Cardiovasc Disord..

[18] Lau CW. [劉卓賢]. (2018). Effectiveness and safety of new oral anticoagulants in patients with nonvalvular atrial fibrillation. (Thesis). University of Hong Kong, Pokfulam, Hong Kong SAR.

[19] Lip GY, Nieuwlaat R, Pisters R, Lane DA, Crijns HJ (2010). Refining clinical risk stratification for predicting stroke and thromboembolism in atrial fibrillation using a novel risk factor-based approach: the euro heart survey on atrial fibrillation. Chest.

[20] Hindricks G, Potpara T, Dagres N, et al. 2020 ESC Guidelines for the diagnosis and management of atrial fibrillation developed in collaboration with the European Association of Cardio-Thoracic Surgery (EACTS) [published online ahead of print, 2020 Aug 29]. Eur Heart J. 2020;ehaa612. 10.1093/eurheartj/ehaa612

[21] Joundi RA, Cipriano LE, Sposato LA, Saposnik G, Stroke Outcomes Research Working Group (2016). Ischemic Stroke Risk in Patients With Atrial Fibrillation and CHA2DS2-VASc Score of 1: Systematic Review and Meta-Analysis. Stroke..

[22] Levey AS, Stevens LA, Schmid CH (2009). A new equation to estimate glomerular filtration rate [published correction appears in Ann Intern Med. 2011 Sep 20;155(6):408]. Ann Intern Med..

[23] Schulman S, Kearon C (2005). Subcommittee on Control of Anticoagulation of the Scientific and Standardization Committee of the International Society on Thrombosis and Haemostasis. Definition of major bleeding in clinical investigations of antihemostatic medicinal products in non-surgical patients. J Thromb Haemost..

[24] Press Releases: CUHK Advocates Atrial Fibrillation Screening and Drug Education to Reduce Risk of Stroke among Elderly. Dated 27 January 2015. Hong Kong SAR: Faculty of Medicine of the Chinese University of Hong Kong (CUHK).

[25] Pugh D, Pugh J, Mead GE (2011). Attitudes of physicians regarding anticoagulation for atrial fibrillation: a systematic review. Age Ageing.

[26] Hess PL, Mirro MJ, Diener HC (2014). Addressing barriers to optimal oral anticoagulation use and persistence among patients with atrial fibrillation: Proceedings, Washington, DC, December 3–4, 2012. Am Heart J.

[27] Brandes A, Smit MD, Nguyen BO, Rienstra M, Van Gelder IC (2018). Risk Factor Management in Atrial Fibrillation. Arrhythm Electrophysiol Rev.

[28] Lip GYH, Keshishian A, Li X (2018). Effectiveness and Safety of Oral Anticoagulants Among Nonvalvular Atrial Fibrillation Patients [published correction appears in Stroke. 2020 Feb;51(2):e44] [published correction appears in Stroke. 2020 Apr;51(4):e71]. Stroke..

[29] Gieling E, de Vries F, Williams R (2019). Mortality risk in atrial fibrillation: the role of aspirin, vitamin K and non-vitamin K antagonists. Int J Clin Pharm.

